# Persistence of tungsten oxide particle/fiber mixtures in artificial human lung fluids

**DOI:** 10.1186/1743-8977-7-38

**Published:** 2010-12-02

**Authors:** Aleksandr B Stefaniak

**Affiliations:** 1 National Institute for Occupational Safety and Health, Centers for Disease Control and Prevention, Morgantown, WV 26505 USA

## Abstract

**Background:**

During the manufacture of tungsten metal for non-sag wire, tungsten oxide powders are produced as intermediates and can be in the form of tungsten trioxide (WO_3_) or tungsten blue oxides (TBOs). TBOs contain fiber-shaped tungsten sub-oxide particles of respirable or thoracic size. The aim of this research was to investigate whether fiber-containing TBOs had prolonged biodurability in artificial lung fluids compared to tungsten metal or WO_3 _and therefore potentially could pose a greater inhalation hazard.

**Methods:**

Dissolution of tungsten metal, WO_3_, one fiber-free TBO (WO_2.98_), and three fiber-containing TBO (WO_2.81_, WO_2.66_, and WO_2.51_) powders were measured for the material as-received, dispersed, and mixed with metallic cobalt. Solubility was evaluated using artificial airway epithelial lining fluid (SUF) and macrophage phagolysosomal simulant fluid (PSF).

**Results:**

Dissolution rates of tungsten compounds were one to four orders of magnitude slower in PSF compared to SUF. The state of the fiber-containing TBOs did not influence their dissolution in either SUF or PSF. In SUF, fiber-containing WO_2.66 _and WO_2.51 _dissolved more slowly than tungsten metal or WO_3_. In PSF, all three fiber-containing TBOs dissolved more slowly than tungsten metal.

**Conclusions:**

Fiber-containing TBO powders dissolved more slowly than tungsten metal and WO_3 _powders in SUF and more slowly than tungsten metal in PSF. Existing pulmonary toxicological information on tungsten compounds indicates potential for pulmonary irritation and possibly fibrosis. Additional research is needed to fully understand the hazard potential of TBOs.

## Introduction

Tungsten is a naturally occurring element that has industrial value in the form of its metal, oxide, and salts. One estimate suggests that up to 800,000 workers may be exposed to tungsten compounds in the United States [1]. One common application of tungsten metal is non-sag wire for light bulb filaments [2]. The production of non-sag wire involves calcination of ammonium paratungstate to yield tungsten oxide powder intermediates which are subsequently reduced under controlled conditions to yield tungsten metal powder for making wire filaments. The type of tungsten oxide intermediate produced depends on the calcination conditions, including furnace atmosphere. Yellow tungsten trioxide (WO_3_) is produced if sufficient air is supplied whereas tungsten blue oxides (TBOs) having varying chemical and physical properties are produced under non-equilibrium conditions when air is excluded. TBO is not a well-defined compound; rather, it is a blue-colored mixture of crystalline hexagonal tungsten bronzes, WO_3_, and various non-stoichiometric tungsten sub-oxides and an amorphous oxide [2,3].

During production of TBOs, aerosols are generated which contain tungsten sub-oxides with fiber-shaped morphology. In a Swedish tungsten refining and manufacturing facility, airborne tungsten oxide fibers had respirable size, length (L) ≥ 5 μm, diameter (D) ≤ 3 μm, and aspect ratio = L/D (AR) ≥ 5:1; the physical diameter of most fibers was ≤0.3 μm [4]. Both single fibers and aggregates of fibers were observed in Swedish workplace atmospheres [4-6]. In the United States, greater than 95% of airborne tungsten-containing fibers collected during tungsten refining and manufacturing and hard metal production had thoracic size (i.e., aerodynamic diameter (D_ae_) ≤ 10 μm) [1,7].

The toxicity of fibers is influenced by their dimension, durability, and dose (at the target tissue). Fiber dimensions determine the extent to which fibers are inhaled, retained, and cleared by the lung. In humans engaged in mouth breathing, deposition in the alveolar region of the lung is maximal for fibers having D_ae _of 2 μm and AR of 3 to 20. Biopersistence is determined by the rate at which fibers are physically cleared from the lung by mechanical action (macrophage- or mucocilliary-mediated) and the rate at which they are dissolved (i.e., biodurability) in lung fluids [8,9]. Fibers longer than 20 μm that deposit on the surface of conducting airways are cleared by mechanical (i.e., mucociliary) action. Because fibers longer than 20 μm that deposit in the unciliated terminal bronchioles or alveoli are not cleared from their deposition site by mucociliary action and probably cannot be engulfed and cleared by a single macrophage, they will likely remain in the lung if biodurable. Engulfment by macrophages of fibers with length between 5 and 20 μm may inhibit macrophage mobility in a graded fashion. Mechanical clearance of fibers shorter than 5 μm by lung alveolar macrophages is probably not influenced by fiber length [8,9]. Fiber chemistry, in part, determines biodurability, as does the lung fluid pH [8,9].

Keith et al. [10], in a U.S. Agency for Toxic Substances and Disease Registry (ATSDR) literature review, has reported the health effects from inhalation of tungsten compounds which has focused almost exclusively on non-fibrous forms. In one study, rats that inhaled tungsten carbide or were intratracheally instilled with tungsten metal or WO_3 _developed mild pulmonary fibrosis [10]; another rat intratrachial instillation study reported that WO_3 _did not affect release of biomarkers linked to fibrosis [11]. Aamodt [12] exposed beagle dogs by nose-only inhalation to WO_3 _particles and determined that 33% of the lung-deposited dose entered the systemic circulation, either via dissolution of particles in lung fluid or translocation to the systemic circulation by alveolar macrophages. In some workers exposed to mixed dusts containing tungsten, the tungsten-containing particles that deposit in the alveolar region of the lung persist for years [13]. Workers in the cemented tungsten carbide industry can be exposed to several tungsten compounds, including the metal, carbide, and tungstenate which vary in their solubility in the body [14]. Only one study investigated tungsten oxide fibers. In this *in vitro *study, laboratory-prepared tungsten oxide fibers, intended to mimic TBO materials, were observed to generate free radicals which could potentially contribute to development of pulmonary fibrosis [15]. Thus, the potential for adverse health effects following inhalation of fiber-shaped tungsten oxide particles is not understood. Given the role of fiber-shaped particles in lung toxicity we investigated whether fiber-containing TBOs are more durable in artificial lung fluids than tungsten metal or WO_3 _and therefore potentially could pose a greater inhalation hazard.

## Materials and methods

### Study overview

The objective of this study was to evaluate whether TBO fibers had prolonged biodurability in artificial lung fluids compared to tungsten metal or WO_3_. Hence, the dissolution of each tungsten oxide powder was measured in the "as-received" (aggregated), "dispersed" (individual particles), and mechanically mixed (with cobalt) states using two different artificial lung fluids. Dissolution of a pure fine tungsten metal and a fine cobalt metal obtained from a hard metal producer [16] were also measured to benchmark durability of the tungsten oxides.

Fibers may induce different pathological effects for aggregated and dispersed forms of the same material. Shvedova et al. [17] reported that aggregated single-walled carbon nanotubes (SWCNT) were associated with induction of granulomas in mouse lung (dosing by pharyngeal aspiration), but dispersed SWCNT were associated with diffuse interstitial fibrosis and alveolar wall thickening. McKernan et al. [1] detected tungsten-containing fibers in a hard metal production facility, indicating potential for exposure to these airborne fibers beyond the intermediate stages of tungsten metal production. The presence of cobalt is known to enhance the toxicity of tungsten carbide by generating larger amounts of free radicals than equivalent masses of cobalt or tungsten carbide alone [18]. Thus, we measured dissolution for each aggregated powder mixed with cobalt at a ratio of 95:5 by weight. These admixtures do not represent any specific industrial powder formulations but were created to understand the co-dissolution of tungsten in the presence of cobalt.

### Study powders

Five bulk tungsten oxide powders were provided for study by an industrial producer of non-sag tungsten metal wire: yellow WO_3 _and the TBOs WO_2.98_, WO_2.81_, WO_2.66_, and WO_2.51_. The only defined tungsten oxide is WO_3_; the TBOs were manufactured under non-equilibrium conditions and the oxidative states of these powders are average values. The WO_2.98 _is crystallographically WO_3_, but has oxygen defects that impart a blue-color to the material. WO_2.81 _is a mixture of WO_2.90 _and WO_2.72_, whereas both WO_2.66 _and WO_2.51 _are mixtures of varying proportions of WO_2.72 _and WO_2_. The WO_3 _and WO_2.98 _powders were examined microscopically as described below and consisted of isometric-shaped particles and accounted for approximately 80% of this manufacturer's production. The WO_2.81_, WO_2.66_, and WO_2.51 _powders were examined microscopically and were mixtures of isometric and fiber particle morphologies and accounted for the remaining 20% of industrial production.

### Study powder characterization

**Figure 1:**
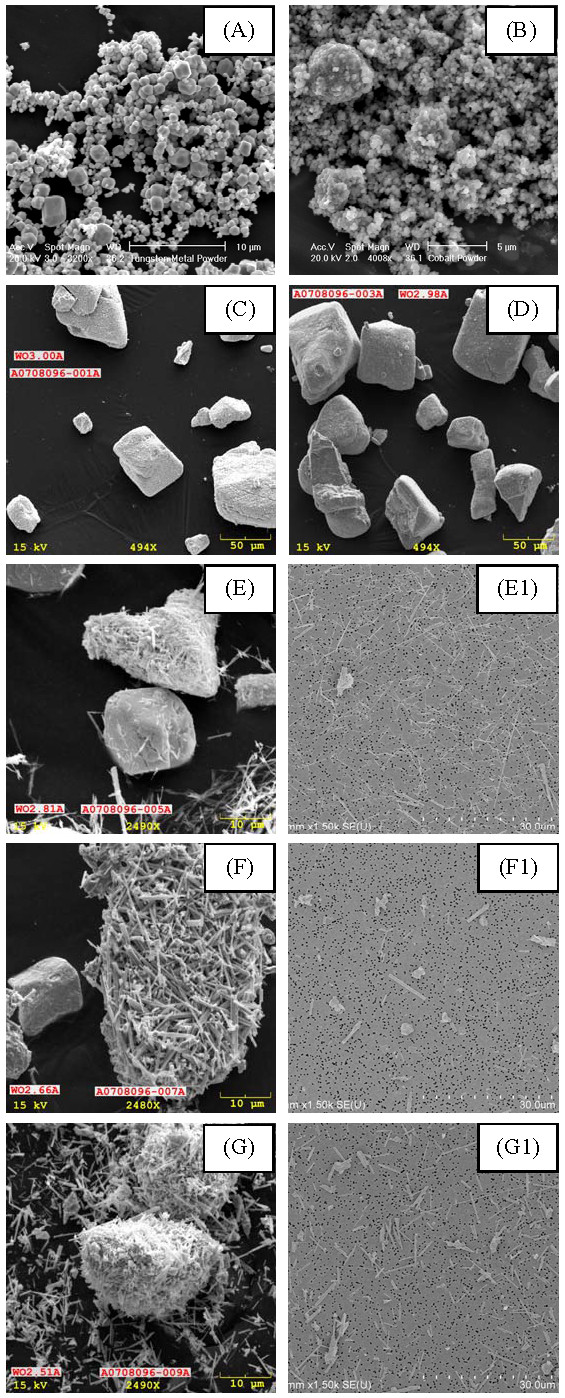
**Scanning electron micrographs of study powders: "as-received" (A) W metal, (B) Co metal, (C) WO_3_, (D) WO_2.98_, (E) WO_2.81_, (F) WO_2.66_, and (G) WO_2.51 _powders**. The W and Co powders were agglomerates of primary particles, the WO_3 _and WO_2.98 _powders were cube-shaped isometric particle morphology, and the WO_2.81_, WO_2.66_, and WO_2.51 _powders were mixtures of fiber- and cube-shaped particle morphologies; "dispersed" (E1) WO_2.81_, (F1) WO_2.66_, and (G1) WO_2.51 _powders illustrating individual fiber morphology. Note that the scale bars differ among images.

A field emission scanning electron microscope (SEM; model S-4800, Hitachi, Tokyo, Japan) was used to evaluate morphology, aggregation state, and external dimensions of the as received and dispersed powders (see Figure 1). Powders were dispersed using distilled water and deposited onto a 0.025 μm pore-size nitrocellulose filter [19]. Helium pycnometry was used to determine powder density by gas displacement (AccuPyc II 1340 Analyzer, Micromeritics, Norcross, GA). Nitrogen gas adsorption was used to determine powder specific surface area (SSA) using a multipoint Brunauer, Emmett, and Teller (BET) instrument (Quadrasorb surface area analyzer, Quantachrome, Boynton Beach, FL). Prior to analysis, powders were outgassed under vacuum (0.013 torr) at 200°C overnight to remove moisture [20].

The U.S. National Institute for Occupational Safety and Health (NIOSH) Method 7400 with "B" counting rules was used to count tungsten-containing fibers, i.e., L > 5 μm, D < 3 μm, and AR of ≥5:1 [1,7]. The relative mass fractions of isometric- and fiber-shaped particles in each powder was calculated from measures of 150 to 300 particle external dimensions in 27 to 50 microscope fields per material and the measured density. The orientation-averaged fiber D_ae _was calculated as described by Griffiths and Vaughn [21]. For isometric-shaped particles, D_ae _was calculated assuming a dynamic shape factor (χ) of 1.08 for cube-shaped WO_3 _and WO_2.98 _particles and χ of 1.00 for spherical tungsten and cobalt particles [22].

Total C, H, and N impurity content [23,24] and S impurity content [25] of powders were determined by combustion by a commercial laboratory (Galbraith Laboratories, Knoxville, TN). Oxygen content was assayed by pyrolysis (LECO Corp, St. Joseph, MI). Inductively coupled plasma (ICP)-atomic emission spectroscopy was used to quantify metal impurities in accordance with NIOSH Method 7303: Elements by ICP [26]. X-ray photoelectron spectroscopy was used to evaluate powder surface (top 20 Å) chemistry (McCrone Associates, Westmont, IL).

### Artificial Lung Fluid Dissolution Studies

Dissolution of triplicate samples of each powder was evaluated using a static technique [27]. Known masses (in the range 10 to 100 mg) of each study powder were determined gravimetrically using a calibrated five-place balance (Model AX 205, Mettler Toledo, Greifensee, Switzerland) capable of reading to 10 μg. Each static dissolution chamber was placed in a separate polypropylene cup, immersed in 80 mL of artificial lung fluid, covered with a lid having two holes to allow gas exchange, and maintained at 37 °C throughout an experiment.

Serum ultrafiltrate (SUF) was used to model dissolution in lung airway epithelial lining fluid [28]. This solvent consists of chloride (217.5 mM), sodium (145.7 mM), ammonium (100.0 mM), calcium (29.4 mM), bicarbonate (27.0 mM), cysteine (1.1 mM), phosphate (1.0 mM). sulfate (0.6 mM), citrate (0.2 mM), and diethylenetriaminepentaacetic acid (2 mM), the latter to prevent formation of insoluble phosphate precipitates. The pH of SUF was maintained at 7.4 ± 0.1 by blanketing the headspace in each sample with 5% CO_2 _and 95% air. Masses of dissolved tungsten were measured after 1, 3, 6, 12, 24, 36, 48, and 72 hours to mimic the residence time of particles in extracellular lung fluid [29]. Phagolysosomal simulant fluid (PSF) buffered with 0.02 M potassium hydrogen phthalate was used to model dissolution in lung alveolar macrophages [30]. This solvent consists of sodium (116.8 mM), chloride (114.2 mM), potassium (25.0 mM), glycine (6.0 mM), phosphate (1.0 mM), sulfate (0.5 mM), and calcium (0.2 mM). The pH of PSF remained at 4.5 ± 0.1 throughout experiments. Masses of dissolved tungsten were measured at 1, 4, 8, 12, 24, and 72 hours, and then twice weekly thereafter to 31 days to mimic the approximate lifespan of an alveolar macrophage [31]. In pilot studies, alkylbenzyldimethylammonium chloride, added to artificial lung fluids to prevent mold growth, inhibited dissolution of some tungsten oxide powders and was omitted from the full-scale experiments. Rather, mold growth was prevented by using sterile labware and laboratory practices.

Experimental and quality control samples were analyzed using U.S. Occupational Safety and Health Administration Method 213: Tungsten and Cobalt in Workplace Atmospheres: inductively coupled plasma analysis [32]. Analysis of dissolved tungsten was performed using inductively coupled plasma-atomic emission spectroscopy (ICP-AES) without sample digestion. The analytical limit of detection (LOD) for tungsten in SUF was 0.03 mg L^-1 ^and the limit of quantification (LOQ) was 0.11 mg L^-1^. For PSF, the tungsten LOD was 0.08 μg L^-1 ^and the LOQ was 0.27 μg L^-1^. Undissolved particulate in the dissolution chambers exposed to SUF and PSF were digested and masses of tungsten quantified using ICP-AES; the tungsten LOD was 0.1 μg sample^-1 ^and the LOQ was 0.43 μg sample^-1^.

### Data analyses

Dissolution parameters for isometric-shaped powders (W, WO_3 _and WO_2.98_) were calculated using a surface-area-limited dissolution model [33]. Dissolution parameters for fiber-containing powders (WO_2.81_, WO_2.66_, and WO_2.51_) were calculated assuming constant dissolution velocity [34]:

(1) $$1-{\left(\frac{M}{M_{0}}\right)}^{\frac{1}{2}}=\frac{2kt}{d_{0}\rho }.$$

where $\frac{M}{M_{0}}$ is the mass fraction of material remaining, t is time (days), d_0 _is the average initial fiber diameter (μm) from SEM analysis, ρ is the measured fiber density (g/cm^3^), and *k *is the chemical dissolution rate constant [g/(cm^2^·day)]. Values of $\text{1}-{\left(\frac{M}{M_{0}}\right)}^{\frac{1}{2}}$ were plotted versus time and non-linear least squares regression models used to estimate the best fit line for the data. The slope value, b, for this line was used to calculate t_1/2 _(i.e., -0.693/b) and to estimate *k *(i.e., b/SSA).

**Figure 2:**
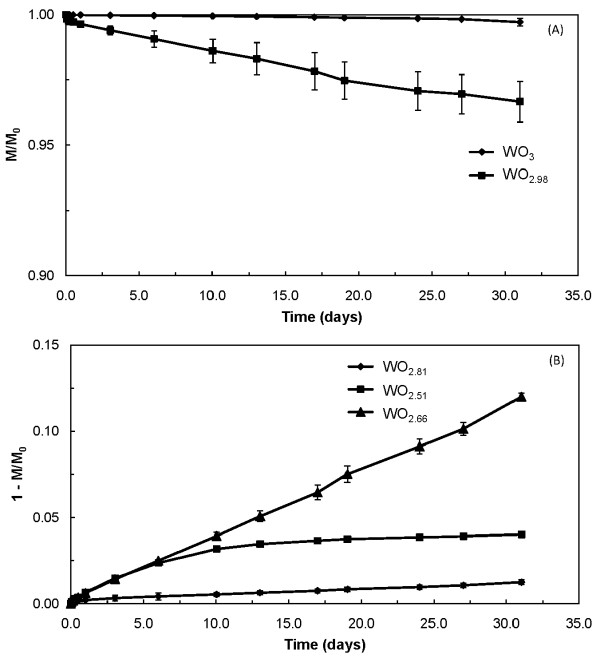
**Plots of tungsten oxide powder dissolution for the as-received state in phagolysosomal simulant fluid**. (A) isometric-shaped WO_3 _and WO_2.98 _powders illustrating single-phase dissolution that was best described by a single negative exponential. Dissolution parameters were calculated using a surface-area-limited dissolution model [33]. Note that the y-axis scale is mass fraction remaining (M/M_0_) and plotted values from 0.90 to 1.0 to illustrate the shape of the curves. (B) fiber-containing WO_2.81_, WO_2.66_, and WO_2.51 _powders; WO_2.81 _and WO_2.51 _illustrate biphasic dissolution that was described by two negative exponentials (the first for the initial rapid dissolution phase and the second for the latter long-term phase) whereas WO2.66 was best described by a single negative exponential. Dissolution parameters for the fiber-containing materials were calculated using a constant dissolution velocity model [34]. Note that the y-axis is 1 - (M/M_0_) and plotted values range from 0.0 to 0.15 to illustrate the shapes of the curves; hence, the fraction of material dissolved during the initial phase was low, usually less than 2% of the total.

For WO_2.81 _and WO_2.51 _in PSF, dissolution data were best described by two negative exponentials, i.e., both a shorter term and a longer term dissolution behavior (see Figure 2B). For these specific cases, a two-part segmented regression model was used to determine the point at which the shorter term phase ended and the longer term phase began [35].

Values of $\text{1}-{\left(\frac{M}{M_{0}}\right)}^{\frac{1}{2}}$ were plotted versus time; however, only the linear portion of the segmented regression, which corresponded to the longer-term dissolution phase was used to calculate dissolution parameters. The slope of the best fit line from the plotted data, b, was used to calculate t_1/2 _and *k *as noted above.

Analysis of variance F-statistics were used to identify overall differences in dissolution half-times and mean rate constants among materials. Tukey's test was used to identify differences between paired means.

## Results

Figure 1 illustrates the morphology of the tungsten oxide study powders. The W and Co powders were both clusters of spherical primary particles. In the as-received state, the WO_3 _and WO_2.98 _powders were large, compact, mostly cube-shaped particles. The WO_2.81_, WO_2.66_, and WO_2.51 _powders were mixtures of compact cube-shaped and fiber-shaped particles. Dispersed WO_2.81 _and WO_2.51 _both contained long, thin fibers and cube-shaped particles, whereas WO_2.66 _contained short, cylinder-shaped and cube-shaped particles.

Table 1 summarizes the physical properties of the study powders. Measured density values for the well-defined WO_3.00 _and W were near theoretical values. The measured density value for Co was slightly lower than theoretical due to the presence of less dense cobalt oxide on particle surfaces as previously determined using Auger spectroscopy [36]. For the TBO powders, density generally increased as powder oxygen content (density 1.3 g/cm^3^) decreased relative to the tungsten content (density 19.2 g/cm^3^) per unit volume of powder. SSA, which depends on particle diameter, density, and surface characteristics (roughness, pores, etc), varied by up to a factor of 15 among powders. Among the WO_2.81_, WO_2.66_, and WO_2.51 _powders, geometric mean (GM) physical fiber lengths were 6 to 8 μm and diameters were ≤0.5 μm. The WO_2.81 _and WO_2.51 _powders were 80% fibers by mass. In contrast, for WO_2.66 _powder, many particles were cylinder-shaped, but only 38% met the definition for a fiber using NIOSH B counting rules.

**Table 1:** Physical properties of study powders.

			D_phys _(μm)^a^		L_phys _(μm)		**L**_**phys**_**/D**_**phys**_	**D**_**ae **_**(μm)**		**Mass (%)**^**b**^	
							
Powder	Density (g/cm^3^)	SSA (m^2^/g)	AM ± SD	GM (GSD)	AM ± SD	GM (GSD)	GM	AM ± SD	GM (GSD)	I	F
WO_3_	7.2 ± 0.0	0.75 ± 0.03	36.2 ± 20.4	31.1 (1.8)	--^c^	--	--	94 ± 53	80.4 (1.8)	100	0
WO_2.98_	7.0 ± 0.0	11.10 ± 0.76	37.5 ± 26.0	29.8 (2.0)	--	--	--	95 ± 66	75.8 (2.0)	100	0
WO_2.81_	7.1 ± 0.0	2.85 ± 0.02	0.3 ± 0.3	0.2 (2.0)	8.8 ± 5.6	7.8 (1.6)	38	1.7 ± 1.4	1.4 (1.8)	21	79
WO_2.66_	8.2 ± 0.0	1.50 ± 0.07	0.5 ± 0.1	0.5 (1.1)	6.3 ± 0.7	6.3 (1.1)	12	3.1 ± 0.3	3.1 (1.1)	62	38
WO_2.51_	7.7 ± 0.1	2.10 ± 0.02	0.5 ± 0.2	0.5 (1.5)	7.6 ± 3.0	7.2 (1.4)	15	3.0 ± 0.9	2.9 (1.4)	21	79
W	19.2 ± 0.1	0.68 ± 0.09	2.5 ± 1.9	2.0 (2.0)	--	--	--	11.1 ± 8.1	8.8 (2.0)	100	0
Co	8.2 ± 0.1	7.30 ± 0.24	2.4 ± 1.7	1.9 (1.9)	--	--	--	6.8 ± 4.9	5.5 (1.9)	100	0

^a ^D_phys _= physical diameter; L_phys _= physical length; D_ae _= aerodynamic diameter. D_ae _values in the Table are orientation-averaged values for the fiber-shaped fractions of the WO_2.81_, WO_2.66_, and WO_2.51 _powders). Calculated D_ae _values for the isometric-shaped particle fraction of the WO_2.81_, WO_2.66_, and WO_2.51 _powders are 2.5 ± 1.5, 7.5 ± 6.0, and 3.7 ± 1.8 μm, respectively.

^b ^Particles having isometric (I)- or fiber (F)-shaped morphology

^c ^-- Not applicable for materials of isometric morphology only

Tungsten oxide powder surfaces were composed of tungsten and oxygen with minor amounts of carbon, which is consistent with tungsten oxides. Measured oxygen content of the well-defined WO_3 _material was greater than 95% of that predicted from the formula weight. The measured oxygen contents of the WO_2.98_, WO_2.81_, WO_2.66_, and WO_2.51 _powders were 19 ± 3%, 19 ± 0%, 16 ± 1%, and 16 ± 4%, respectively, which are consistent with the masses expected from the average stoichiometries of the powder constituents. The oxygen content of the tungsten metal study powder was <0.3%. Levels of impurities in the tungsten oxide materials were either below analytical LODs or ≤0.001% by weight (Sb, Ba, Cr, Cu, Fe, Se, and V).

### Dissolution results

In general, tungsten dissolution from each study powder consisted of a single phase that was best described by a single negative exponential. For a few powder/solvent combinations, dissolution was biphasic, consisting of an initial rapid dissolution phase followed by a long-term dissolution phase; these data were best described by two negative exponentials. The fraction of material dissolved during the initial phase was low, usually less than 2% of the total, so reported dissolution rates refer only to the long-term phase (see Figure 2).

#### Artificial airway epithelial lining fluid

Table 2 summarizes the fitted long-term dissolution parameters for the tungsten oxide powders in three states (as-received, dispersed, and mixed with cobalt) for a 72-hour immersion in SUF. In the as-received state, values of t_1/2 _for the fiber-containing WO_2.51 _and WO_2.66 _powders were longer and their corresponding *k *values were slower compared to all other powders (p < 0.05). In the dispersed state, values of t_1/2 _for the fiber-containing WO_2.51 _and WO_2.66 _powders were longer than for the other tungsten oxide powders (p < 0.05). Dispersed fiber-containing WO_2.81_, WO_2.66_, and WO_2.51 _powders had slower *k *values than the isometric-shaped WO_3 _and WO_2.98 _powders (p < 0.05). Dissolution of cobalt in SUF was biphasic and independent of the presence of any tungsten oxide powder (data not shown). The dissolution half-time for WO_2.98 _was shortest among all tungsten oxide/cobalt powder mixtures (p < 0.05). There were no differences in *k *values among the tungsten oxide powders mixed with cobalt.

**Table 2:** Long-term dissolution parameters (mean ± standard deviation) for tungsten-containing materials after immersion in artificial airway epithelial lining fluid (pH 7.4) for 72 hours

	As Received		Dispersed		Mixture with Cobalt	
			
Powder	**t**_**1/2 **_**(days)**	***k *[g/(cm**^**2**^**·day)]**	**t**_**1/2 **_**(days)**	***k *[g/(cm**^**2**^**day)]**	**t**_**1/2 **_**(days)**	***k *[g/(cm**^**2**^**·day)]**
WO_3_	4 ± 1	2.5 ± 0.3 × 10^-5^	11^a^	0.9 × 10^-5^	79 ± 23	1.3 ± 0.4 × 10^-6^
WO_2.98_	1 ± 0	6.0 ± 1.2 × 10^-6^	1 ± 0	6.0 ± 2.3 × 10^-6^	10 ± 2	6.5 ± 1.6 × 10^-7^
WO_2.81_	11 ± 2	2.3 ± 0.3 × 10^-6^	13 ± 1	1.9 ± 0.1 × 10^-6^	71 ± 6	3.5 ± 0.3 × 10^-7^
WO_2.66_^b^	56 ± 10	8.4 ± 1.7 × 10^-7^	41 ± 2	1.1 ± 0.1 × 10^-6^	72 ± 22	6.8 ± 1.8 × 10^-7^
WO_2.51_	81 ± 4	4.1 ± 0.2 × 10^-7^	50 ± 14	6.9 ± 1.7 × 10^-7^	89 ± 13	3.8 ± 0.6 × 10^-7^
W	19 ± 2	5.4 ± 0.5 × 10^-6^	--^c^	--	--	--

^a ^Result for WO_3 _in dispersed state is for n = 1 sample

^b ^Values for t_1/2 _(days) and *k *[g/(cm^2^·day)] calculated using surface-area-limited dissolution model: 30 ± 6, 1.6 ± 0.3 × 10^-6 ^(as-received); 22 ± 2, 2.1 ± 0.2 × 10^-6 ^(dispersed); 37 ± 11, 1.3 ± 0.3 × 10^-6 ^(mixture).

^c ^Not applicable for the tungsten metal samples

For a given fiber-containing tungsten oxide powder, *k *values in SUF did not differ among the as-received, dispersed, and mixture states. Dissolution rates (*k*-values) for the isometric-shaped WO_3 _and WO_2.98 _powders did not differ between the as-received and dispersed states; however, in the presence of cobalt their rates were slower.

#### Artificial alveolar macrophage phagolysosomal fluid

Table 3 summarizes the fitted long-term dissolution parameters for the tungsten-containing powders during 31 days of immersion in PSF. In general, dissolution rates of tungsten oxides in PSF were one to four orders of magnitude slower than in SUF. In the as-received state, t_1/2 _ranged from 97 days (W) to 9893 days (WO_3_) with the rank order (from longest to shortest) being: WO_3 _≈ WO_2.98 _≈ WO_2.81 _≈ WO_2.51 _> WO_2.66 _> W (p < 0.05). The corresponding rank order of *k *values (from fastest to slowest) was: W > WO_2.66 _> WO_3 _≈ WO_2.98 _≈ WO_2.81 _≈ WO_2.51 _(p < 0.05). In the as-received state, the *k *value for W metal was faster than that for WO_2.66 _and the rates for these two compounds were both faster than the rates for the remaining tungsten oxides (p < 0.05). In the dispersed state, t_1/2 _values ranged from hundreds to thousands of days; WO_2.66 _was significantly faster than all other oxide powders. The dissolution rate for dispersed fiber-containing WO_2.66 _powder was faster than rates for all other dispersed tungsten oxide powders (p < 0.05). There was no difference in *k *values among the dispersed WO_3_, WO_2.98_, WO_2.81_, and WO_2.51 _powders. In the powder mixtures, dissolution of cobalt was independent of tungsten oxide (data not shown). When mixed with cobalt, t_1/2 _values for the tungsten oxide powders ranged from 200 to 8000 days; WO_2.66 _was significantly faster than all other oxide powders. The corresponding *k*-value for WO_2.66 _powder was faster than all other tungsten oxide powders (p < 0.05).

**Table 3:** Long-term dissolution parameters (mean ± standard deviation) for tungsten-containing materials after immersion in artificial lung alveolar macrophage phagolysosomal fluid (pH 4.5) for 31 days

	As Received		Dispersed		Mixture with Cobalt	
			
Powder	**t**_**1/2 **_**(days)**	***k *[g/(cm**^**2**^**·day)]**	**t**_**1/2 **_**(days)**	***k *[g/(cm**^**2**^**·day)]**	**t**_**1/2 **_**(days)**	***k *[g/(cm**^**2**^**·day)]**
WO_3_	9893 ± 2549	9.8 ± 2.9 × 10^-9^	21541 ± 1890	4.3 ± 0.4 × 10^-9^	8052 ± 2458	1.1 ± 0.4 × 10^-8^
WO_2.98_	656 ± 154	9.9 ± 2.6 × 10^-9^	929 ± 45	6.8 ± 0.3 × 10^-9^	655 ± 72	9.6 ± 1.0 × 10^-9^
WO_2.81_	2218 ± 84	1.1 ± 0.0 × 10^-8^	2207 ± 144	1.1 ± 0.1 × 10^-8^	2084 ± 102	1.2 ± 0.1 × 10^-8^
WO_2.66_^a^	181 ± 7	2.6 ± 0.1 × 10^-7^	214 ± 6	2.2 ± 0.1 × 10^-7^	218 ± 17	2.1 ± 0.2 × 10^-7^
WO_2.51_	2455 ± 422	1.4 ± 0.2 × 10^-8^	2915 ± 414	1.1 ± 0.2 × 10^-8^	1343 ± 252	2.5 ± 0.5 × 10^-8^
W	97 ± 17	1.1 ± 0.2 × 10^-6^	--^b^	--	--	--

^a ^Values for t_1/2 _(days) and *k *[g/(cm^2^·day)] calculated using surface-area-limited dissolution model: 87 ± 4, 5.3 ± 0.2 × 10^-7 ^(as-received); 106 ± 3, 4.4 ± 0.1 × 10^-7 ^(dispersed); 101 ± 8, 4.6 ± 0.4 × 10^-7 ^(mixture).

^b ^Not applicable for the tungsten metal samples

For a given tungsten oxide powder, values of t_1/2 _did not differ among states in PSF with the exception of a longer half-time for WO_3 _in the dispersed state than in the as-received and mixed states. For a given powder, its *k*-values did not differ among states in PSF.

## Discussion

The rate at which materials are cleared from the respiratory tract depends upon the location in which the material deposits, the physicochemical form of the material, and the time since deposition [37]. In the present study, dissolution was evaluated for tungsten oxides having varying physicochemical properties in two artificial lung environments, one mimicking the fluid lining the airways (i.e., SUF) and the other mimicking the fluid contained in alveolar macrophage phagolysosomes (i.e., PSF). Clear differences in dissolution behavior were observed among and within tungsten oxide powders in SUF and PSF. Such differences are important for understanding the relative persistence of tungsten oxides in the lung.

Mechanical clearance half-times for durable particles that deposit in the conducting airways are approximately 8 hours (bronchioles to bronchi), 100 minutes (bronchi to extrathoracic airways), and 10 minutes (extrathoracic airways to the gastrointestinal tract) via the mucociliary escalator [37]. In SUF, the as-received and dispersed WO_3 _and WO_2.98 _had the fastest dissolution rates among all the studied powders, though the presence of cobalt slowed dissolution of these materials. Based on the measured surface area and dissolution rates, the amounts of material dissolved from WO_3 _in each of the above-listed clearance half-times would be 6% (8 hours), 1% (100 minutes), and 0.1% (10 minutes), whereas the amounts of material dissolved from WO_2.98 _would be 20% (8 hours), 5% (100 minutes), and 0.5% (10 minutes). Thus, clearance of these materials from the conducting airways would include both mechanical action and dissolution. The WO_3 _and WO_2.98 _powders are crystallographically similar materials and the high solubility of these materials supports the results of Aamodt [12], who observed that approximately one-third of an inhaled WO_3 _particle dose enters systemic circulation. Interestingly, in all states evaluated, WO_2.66 _dissolved faster in SUF than the other fiber-containing samples which may be due to its higher mass fraction of isometric-shaped particles (Table 1). However, in all states evaluated, the fiber-containing tungsten oxide powders dissolved more slowly than the isometric-shaped materials. This suggests that clearance of fiber-containing tungsten oxide powders from the conducting airways would be dominated by mechanical mechanisms with little (<1%) dissolving during a single clearance half-time.

The orientation-averaged D_ae _and aspect ratios of the WO_2.81_, WO_2.66_, and WO_2.51 _fibers indicate that they were respirable, with high probability of deposition in the non-ciliated alveolar region of the lung (Table 1). The dimensions of fibers in these bulk tungsten oxide powders are consistent with previous reports that airborne tungsten oxide fibers in workplace atmospheres had respirable or thoracic size [1,4,7]. Within 24 hours, 90% of particles deposited in the alveolar region of the lung are associated with macrophage cells [29]. The lengths of the WO_2.81_, WO_2.66_, and WO_2.51 _fibers ranged from 6 to 9 μm, which is sufficient to inhibit their clearance from alveoli by macrophages [8,9]. As such, dissolution in macrophage phagolysosomal fluid is likely to play an important role in the clearance of relatively insoluble materials such as TBO fibers that deposit in the alveolar region of the lung.

Often, rate of metal dissolution increases as solvent pH decreases [38]. However, in the current study, dissolution rates of each tungsten oxide material were up to four orders of magnitude slower in PSF than in SUF, indicating potential for long-term retention of tungsten oxides that deposit in the alveolar region of the lung. With regards to dissolution in PSF, the fiber-containing WO_2.81_, WO_2.66_, and WO_2.51 _powders dissolved more slowly in the as-received state than tungsten metal powder, indicating potential for longer persistence in the alveoli. In the dispersed and mixed states, WO_2.66 _powder, but not WO_2.81 _or WO_2.51 _powder, dissolved more rapidly than the other tungsten oxide powders. Overall, these results suggest that fiber-containing tungsten oxides that deposit in the alveolar region of the lung would have similar biopersistence to isometric-shaped WO_3 _and WO_2.98_, but would be more biopersistent than tungsten metal.

The toxicity of tungsten compounds provides limited clues as to potential adverse effects from inhalation of tungsten oxide fibers. Workers that grind cemented tungsten carbides are exposed to mixed dusts containing tungsten, which persists in the alveoli for years [13]. Workers in the cemented tungsten carbide industry are exposed to several tungsten compounds which have a range of solubility in the body [14]. WO_3 _was reported to cause mild pulmonary fibrosis in rats [10], though this effect was not confirmed in another study [11]. Laboratory-prepared TBO materials can generate free radicals at rates higher than asbestos *in vitro*, which could contribute to development of pulmonary fibrosis [15] though it is unknown whether this reaction occurs *in vivo*. Tungsten oxide fibers can be present in workplaces that use cobalt [1] and cobalt is known to enhance the toxicity of some tungsten compounds by generating free radicals [18]. In the current study, the dissolution rates of each fiber-containing tungsten oxide powder did not differ among the as-received, dispersed, and cobalt mixture states. Hence, whether inhaled as tungsten oxide or tungsten oxide/cobalt mixtures, these powders will persist in the alveoli and present opportunity for generation of free radicals. Shvedova et al. [17] reported that dispersed SWCNT were associated with diffuse interstitial fibrosis and aggregated SWCNT induced granulomas in mouse lung. Particle dispersion may variably influence *in vivo *biopersistence in relation to the capacity of macrophages to engulf agglomerated versus individual fibers. In the current study, there were no differences in dissolution rates between as-received and dispersed tungsten oxide fibers. The potential for adverse health effects due to long-term persistence of tungsten oxide fibers in the alveolar region of the lung remains poorly understood.

Finally, it is interesting to note that historically, an early method for producing non-sag tungsten metal wire involved reduction of WO_3_. Later, in the 1970 s, industrial producers began to use TBOs because these materials have higher surface area and therefore are better at absorbing potassium ions, which are important for product performance [2]. While the TBOs are better at absorbing potassium ions, WO_3 _can be made to have the same surface area as TBOs and high-quality wire can be produced using WO_3 _[3]. The WO_3 _material evaluated in this study had a mean equivalent D_ae _of approximately 100 μm, much larger than the D_ae _of the three fiber-containing TBOs. Thus, when inhaled, the WO_3 _material evaluated in this study would be more likely to deposit in the more rapidly cleared upper conducting airways and less likely to be deposited in the more slowly cleared alveolar regions of the lung. Also, the WO_3 _material evaluated in this study was more soluble than the three fiber-containing TBOs evaluated in this study. In light of the measured particle physical characteristics and observed dissolution data, *in vivo *studies to evaluate toxicity of TBOs relative to WO_3 _powder may be worthwhile to determine whether substitution of WO_3 _for TBOs is appropriate.

## Summary

The current study focused on the durability of three fiber-containing tungsten oxide powders in artificial lung fluids and compared their dissolution behavior to tungsten metal and two isometric-shaped tungsten oxide powders. Dissolution rates of tungsten compounds were one to four orders of magnitude slower in PSF compared to SUF. The physical state of the tungsten oxides (as-received versus dispersed) had no effect on dissolution behavior in either SUF or PSF. The presence of cobalt did not influence dissolution of the fiber-containing tungsten oxide powders; however, dissolution rates of WO_3 _and WO_2.98 _in SUF were slowed by cobalt. Compared to tungsten metal powder, a known mild pulmonary irritant, dissolution rates of fiber-containing WO_2.66 _and WO_2.51 _powders were slower in SUF and dissolution rates of all three fiber-containing TBO powders were slower in PSF. The slow dissolution of tungsten oxide fibers, coupled with existing toxicology information on tungsten compounds, suggests additional research is needed to fully understand the hazard potential of these fibers.

## Competing interests

The authors declare that they have no competing interests.

## Authors' contributions

ABS designed the study, coordinated its completion, and drafted the manuscript. The author read and approved the final manuscript.
